# Posterior Single-Window Ultrasound-Guided Cryoneurolysis for Severe Pediatric Spastic Equinovarus: Technical Feasibility and Same-Patient Comparison

**DOI:** 10.3390/reports9030224

**Published:** 2026-07-14

**Authors:** Luigi Di Lorenzo, Hassan Zmerly, Emiliano Agliaroro, Alfonso Maria Forte, Valeria Marinò

**Affiliations:** 1 Istituto Neurologico Mediterraneo Neuromed IRCCS, 86042 Pozzilli, Italya.forte@gruppoforte.it (A.M.F.); valeriamarino.73@gmail.com (V.M.); 2 Department Vita e Salute MED 34, Link Campus University, 44-00165 Rome, Italy; h.zmerly@unilink.it

**Keywords:** cryoneurolysis, spasticity, cerebral palsy, equinovarus, ultrasound-guided intervention, neurorehabilitation, pediatric rehabilitation, tibial nerve, gastrocnemius, soleus

## Abstract

**Background and Clinical Significance:** Severe pediatric spastic equinovarus may significantly impair positioning, orthotic tolerance, hygiene management, caregiver-assisted mobilization, and assisted standing activities. In children with severe cerebral palsy, clinically meaningful outcomes frequently include reduction in caregiver burden and facilitation of daily care rather than restoration of autonomous gait. Ultrasound-guided cryoneurolysis has recently emerged as a minimally invasive option for focal spasticity management, although procedural workflow and tolerability remain challenging in severe deforming patterns. **Case Presentation**: We report a CARE-compliant same-patient bilateral technical comparison in a 9-year-old child with severe spastic cerebral palsy and bilateral dynamic equinovarus refractory to intensive rehabilitation and repeated botulinum toxin treatment. Baseline severity was consistent with GMFCS level IV. One lower limb was treated using the proposed posterior single-window ultrasound-guided cryoneurolysis approach through a single posterior proximal-calf window, whereas the contralateral limb underwent a conventional multi-point supine strategy. The posterior single-window approach enabled sequential targeting of multiple motor branches through a single posterior access corridor under continuous ultrasound guidance. The procedure required approximately 1 mL of 2% lidocaine without additional sedation and was completed in approximately 4 min, whereas the conventional supine strategy required multiple access points, repeated probe repositioning, minimal conscious sedation with midazolam, and approximately 20 min. At follow-up, lower-limb spasticity improved from approximately MAS 3 toward MAS 2, passive ankle angle, measured as the tibia–foot angle with 90° corresponding to the neutral ankle position, improved from approximately 80° to 95°, and semitendinosus-related hypertonia was reduced. Clinically meaningful improvement in positioning, hygiene management, assisted standing, and rehabilitation handling was observed. Caregiver-reported satisfaction and procedural tolerability were qualitatively perceived as better with the posterior single-window approach. **Conclusions**: The proposed posterior single-window cryoneurolysis strategy may represent a technically simplifying and clinically relevant minimally invasive approach for severe pediatric spastic equinovarus. Further prospective studies are required to confirm reproducibility, safety, and long-term outcomes.

## 1. Introduction and Clinical Significance

Spastic equinovarus represents one of the most frequent and disabling lower-limb deformities in children with cerebral palsy and is commonly associated with overactivity of the gastrocnemius–soleus complex and tibialis posterior muscles [1]. In severe forms, the condition may significantly impair positioning, orthotic tolerance, hygiene management, caregiver-assisted mobilization, and standing activities. Functional impairment in severe cerebral palsy is frequently more closely related to neuromotor severity and spasticity burden than to static deformity alone [2]. In children with severe motor impairment, clinically meaningful outcomes frequently extend beyond gait performance and may include facilitation of hygiene, positioning, orthotic tolerance, caregiver-assisted mobilization, and reduction in caregiver burden.

Selective peripheral nerve procedures are increasingly used in focal spasticity management. In particular, diagnostic and therapeutic targeting of tibial motor branches has progressively gained interest for the management of spastic equinovarus patterns in pediatric cerebral palsy [1]. Anatomical ultrasonographic studies demonstrated the importance of selective identification of gastrocnemius, soleus, and tibialis posterior motor branches in order to optimize focal interventions [1]. Although botulinum toxin injections remain a standard treatment option for focal spasticity, repeated procedures, limited duration of effect, procedural discomfort, and sedation requirements may represent important limitations in children with severe deforming patterns and complex motor impairment.

Cryoneurolysis has recently emerged as a minimally invasive treatment option for focal spasticity management [3,4,5,6,7,8,9,10]. The technique induces a reversible axonotmetic lesion while preserving the connective architecture of the nerve, potentially allowing prolonged reduction in neural overactivity with progressive axonal regeneration over time [11]. Following the original description of cryoneurotomy as a minimally invasive treatment strategy for spasticity by Paul Winston and colleagues [6], increasing evidence has progressively supported the role of ultrasound-guided cryoneurolysis in severe focal spasticity involving both upper and lower limbs [3,4,5,6,7,8,9,10]. Compared with chemical neurolysis or surgical neurotomy, cryoneurolysis may offer the potential advantage of prolonged neural modulation while maintaining a minimally invasive and potentially repeatable procedural profile.

Recent reports emphasized the importance of dynamic and multimodal strategies during cryoneurolysis procedures, particularly in patients with severe deforming patterns refractory to conventional treatment [3,4,7,9,10]. Furthermore, recent technical observations suggested that altered functional anatomy in severe chronic spasticity may complicate conventional targeting strategies and require adaptive procedural approaches in order to improve selectivity and reduce complications [12,13]. In severe chronic spasticity, altered muscle architecture, reduced compliance, and procedural discomfort may complicate conventional targeting strategies, particularly during multi-point ultrasound-guided procedures in pediatric patients. Consequently, simplified and ergonomically optimized approaches may represent an important practical advantage in selected severe cases.

The present technical case report was prepared according to the CARE guidelines for case reports [14] and describes a same-patient bilateral comparison between two ultrasound-guided cryoneurolysis strategies in a child with severe spastic cerebral palsy and bilateral dynamic equinovarus. One lower limb was treated using a posterior single-window approach [15], whereas the contralateral limb underwent a conventional multi-point supine strategy [15]. The report focuses not only on technical feasibility and procedural workflow, but also on tolerability, procedural burden, caregiver-centered outcomes, and practical implications for severe pediatric neurorehabilitation. The proposed approach was conceptually inspired by the anatomical targeting philosophy proposed by Alessandro Picelli regarding dynamic ultrasound-guided selective nerve procedures in spasticity management [1,13] and by the minimally invasive cryoneurotomy philosophy described by Paul Winston [6].

## 2. Case Presentation

### 2.1. Patient Information

A 9-year-old child with severe spastic cerebral palsy presented with bilateral dynamic equinovarus associated with marked lower-limb spasticity predominantly involving the gastrocnemius, soleus, tibialis posterior, and bilateral semitendinosus muscles.

The patient demonstrated severe motor impairment with major limitations in positioning tolerance, passive mobilization, hygiene management, assisted standing, and caregiver-assisted transfers. Daily care required extensive caregiver assistance during dressing, hygiene, mobilization, and rehabilitation activities.

The child had previously undergone intensive multidisciplinary rehabilitation treatment including physiotherapy, orthotic management, serial rehabilitation programs, and repeated botulinum toxin injections with only partial and temporary clinical benefit.

Given the severity of the neurological impairment, the primary therapeutic goals were not restoration of autonomous ambulation, but reduction in focal spasticity, facilitation of hygiene and positioning, improvement of passive joint mobility, reduction in caregiver burden, and facilitation of assisted standing and rehabilitation handling.

### 2.2. Clinical Findings

Clinical examination demonstrated severe plantar-flexor overactivity with dynamic equinovarus deformity involving both lower limbs. Marked spasticity predominantly affected the gastrocnemius–soleus complex and tibialis posterior muscles, with associated bilateral semitendinosus involvement.

Baseline functional severity was consistent with Gross Motor Function Classification System (GMFCS) level 4. At clinical examination, lower-limb spasticity was graded approximately as Modified Ashworth Scale (MAS) 3 in the gastrocnemius–soleus complex and posterior muscular chain before treatment, with post-procedural reduction toward MAS 2 at follow-up evaluation. Modified Tardieu assessment demonstrated an R1–R2 difference of approximately 20–30° before treatment, suggesting a substantial dynamic spastic component.

Passive ankle range was measured clinically as the tibia–foot angle using manual goniometry, with 90° corresponding to the neutral ankle position. According to this measurement method, passive ankle angle improved from approximately 70° before treatment to approximately 95° after intervention. Therefore, the reported values should not be interpreted as conventional dorsiflexion degrees above neutral. A clinically significant increase in knee flexion excursion associated with reduction in semitendinosus hypertonia was also observed during assisted mobilization and rehabilitation handling.

Reduced passive ankle dorsiflexion, impaired positioning tolerance, difficulties during hygiene management, and limitations during assisted standing and mobilization were documented during baseline evaluation. The severe spastic pattern significantly interfered with rehabilitation handling and daily caregiver-assisted activities.

### 2.3. Therapeutic Rationale and Intervention

After multidisciplinary evaluation and informed parental consent, bilateral ultrasound-guided cryoneurolysis was proposed as a minimally invasive focal spasticity management strategy.

The rationale for intervention was based on the severity of the deforming spastic pattern, limited benefit obtained from previous botulinum toxin treatment, and the need to reduce procedural burden while improving daily care and rehabilitation management.

Motor targets included the motor branches to the medial and lateral gastrocnemius, soleus, tibialis posterior, and semitendinosus muscles. Target selection was based on the patient’s clinical pattern of spasticity and ultrasound-guided anatomical identification of the corresponding motor branches. The anatomical rationale for selective targeting was based also on previously described ultrasonographic studies regarding tibial motor branch localization in pediatric spastic equinovarus [1].

### 2.4. Technical Procedures

All procedures were performed under sterile conditions using continuous high-frequency ultrasound guidance.

A GE HealthCare Versana Active ultrasound system (GE HealthCare, Chicago, IL, USA) equipped with a high-frequency L9-RS linear transducer (3–12 MHz) was used for all procedures. Under continuous ultrasound guidance, the posterior calf compartments were systematically examined to identify the gastrocnemius, soleus, and tibialis posterior muscular planes, as well as the tibial nerve and its motor branches at the proximal calf level. The target region was selected according to the sonographic relationship between the tibial nerve motor branches and the surrounding muscular compartments. Color Doppler imaging was routinely employed before needle or cryoprobe advancement to identify and avoid adjacent vascular structures.

All procedures were performed under sterile conditions. Local anesthesia consisted of approximately 1 mL of 2% lidocaine infiltrated into the skin and subcutaneous tissue at the planned entry site. No intentional perineural local anesthetic injection was performed prior to cryoneurolysis in order to preserve anatomical visualization and avoid altering procedural assessment.

The cryoprobe was introduced using an in-plane ultrasound-guided technique (GeHealtchare Vertana LapTop Linear Probe L9 USA) and advanced under continuous real-time visualization. Through a single posterior access corridor, the gastrocnemius, soleus, and tibialis posterior motor branches were sequentially targeted by controlled redirection of the cryoprobe without the need for additional skin punctures. The formation of the ice ball was continuously monitored sonographically to ensure accurate coverage of the intended motor branch while minimizing unintended extension toward adjacent non-target tissues.

**Figure 1:**
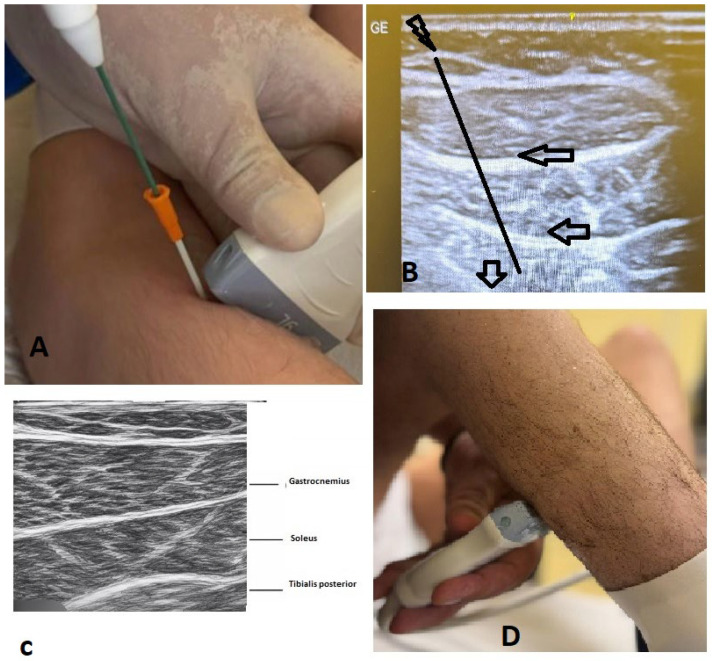
Composite illustration of the posterior single-window ultrasound-guided cryoneurolysis technique. (**A**) Clinical view of the index patient demonstrating the prone position, ultrasound probe orientation, cryoprobe insertion, and the single posterior skin entry used for the posterior single-window approach. (**B**) Representative ultrasound image illustrating the in-plane cryoprobe trajectory. The black line indicates the cryoprobe trajectory, while the arrows identify the probe entry site and the target motor branches within the posterior calf compartment. (**C**) Representative ultrasound image illustrating the sonographic anatomy of the posterior calf muscles (gastrocnemius, soleus, and tibialis posterior). (**D**) Representative clinical positioning of the ultrasound probe during the posterior single-window approach. Panels (**C**) and (**D**) were obtained from another patient with a comparable spastic equinovarus pattern treated using the same posterior single-window technique. Written informed consent for the acquisition and publication of these clinical and ultrasound images was obtained from the patient and/or the patient’s legal guardian, in accordance with institutional policies and the Declaration of Helsinki. These images are included exclusively to better illustrate the sonographic anatomy and technical aspects of the procedure.

Cryoneurolysis was performed using the Cryo-S Painless^®^ cryoneurolysis system (Metrum Cryoflex, Stare Babice, Poland). A 120 mm, 18-gauge cryoprobe was used for all procedures. Each motor branch was treated with one 60 s freeze–thaw cycle according to the manufacturer’s recommendations and the operator’s standard clinical protocol. The term “posterior single-window approach” is used to describe a posterior single-entry ultrasound-guided technique developed in our clinical practice. In this approach, multiple posterior calf motor targets are reached through one skin entry point and one ultrasound-guided posterior anatomical window. The technique was conceptually inspired by selective tibial motor branch targeting principles and minimally invasive ultrasound-guided cryoneurolysis concepts previously described in the literature [1,6,13] and the minimally invasive ultrasound-guided cryoneurolysis technique introduced by Winston et al. [6]. In this approach, multiple posterior calf motor targets are reached through one skin entry point and one ultrasound-guided posterior anatomical window. For clarity, the term “posterior single-window approach” is used throughout the revised manuscript. The patient was positioned prone and a single posterior procedural window was identified at the proximal calf level using ultrasound guidance. Through the same posterior access corridor, sequential ultrasound-guided cryoneurolysis was performed on the motor branches to the medial and lateral gastrocnemius, soleus, tibialis posterior, and semitendinosus muscles by controlled redirection of the cryoprobe without additional skin punctures whenever anatomically feasible (Figure 1).

The approach was conceptually inspired by the anatomical targeting philosophy proposed by Alessandro Picelli for selective tibial motor branch procedures [1] and by the minimally invasive cryoneurotomy concepts introduced by Paul Winston [6].

On the contralateral lower limb, the patient underwent a conventional multi-point supine cryoneurolysis strategy. Selective targeting of soleus motor branches, tibialis posterior-related branches, and lateral gastrocnemius motor branches required multiple access points and repeated probe repositioning maneuvers.

Compared with the posterior single-window strategy, the conventional approach appeared technically more complex, procedurally longer, and significantly less tolerated. Because of procedural discomfort and reduced compliance, minimal conscious sedation with midazolam became necessary. Total procedural duration was approximately 20 min.

The principal technical differences between the two procedures are summarized in Table 1.

### 2.5. Follow-Up and Outcomes

Both procedures were completed without immediate complications or adverse events.

The patient was followed clinically for 3 months after the procedure. Follow-up evaluations were performed at 1 month and 3 months.

At 1-month and 3-month follow-up evaluations, clinically meaningful reduction in lower-limb spasticity was observed, particularly involving the gastrocnemius–soleus complex and posterior muscular chain. Improvement in passive mobilization, positioning tolerance, hygiene management, and assisted standing was documented during clinical examination and caregiver-assisted mobilization.

During the 3-month follow-up period, spasticity did not return to the pre-treatment baseline according to clinical examination.

Although restoration of independent ambulation was not considered a realistic therapeutic goal, reduction in lower-limb spasticity facilitated supported gait training using a bilateral axillary walker.

According to caregivers and rehabilitation staff, the child demonstrated greater lower-limb flexibility and movement freedom compared with previous botulinum toxin treatments. Improvement in rehabilitation handling and standing tolerance was also reported. At follow-up evaluation, reduction in spasticity was clinically associated with improvement in passive ankle mobilization, increased dorsiflexion range, improved knee flexion mobility, and reduced posterior chain stiffness. Improvement in semitendinosus-related hypertonia facilitated caregiver-assisted positioning and rehabilitation handling.

Caregiver-reported satisfaction appeared substantially greater following the single-window approach procedure compared with the conventional multi-point supine strategy. Caregiver feedback was collected qualitatively and was not based on a validated satisfaction scale. Caregivers reported better tolerability and lower perceived procedural burden during the posterior single-window approach compared with the conventional multi-point supine procedure.

Because the two limbs were treated using different procedural strategies, follow-up findings were described separately when clinically appreciable. Both limbs showed reduction in plantar–flexor hypertonia and improved passive mobilization. The limb treated with the posterior single-window approach showed better procedural tolerability during treatment; however, no definitive conclusion regarding superior clinical efficacy between limbs can be drawn from this single-patient observation.

### 2.6. Caregiver Perspective

The caregivers reported marked satisfaction with easier hygiene management, improved dressing and mobilization, reduced lower-limb stiffness, improved positioning, and easier assisted standing.

**Figure 2:**
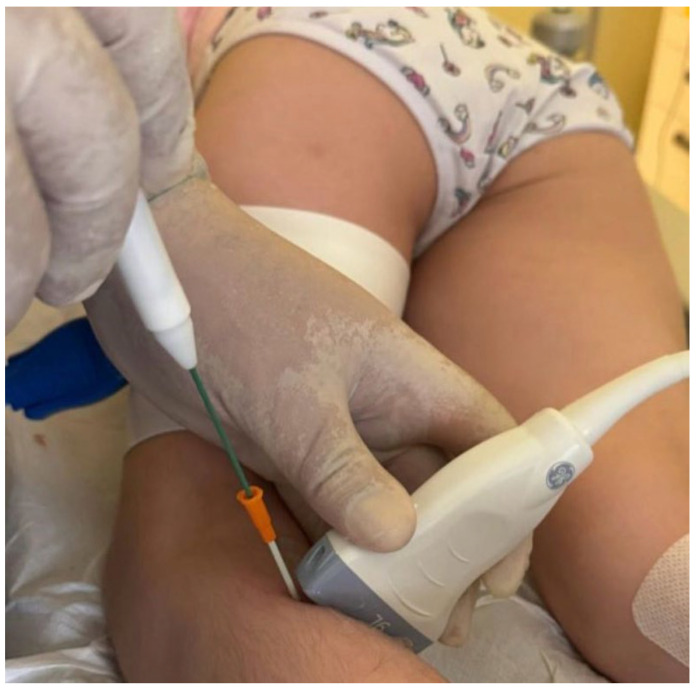
Clinical procedural view of the posterior single-window ultrasound-guided cryoneurolysis approach in a child with severe spastic equinovarus. The procedure was performed through a single posterior access corridor at the proximal calf level under continuous ultrasound guidance. Sequential targeting of multiple motor branches was achieved through controlled cryoprobe redirection within the same anatomical window, facilitating simplified workflow and reduced procedural burden.

Compared with the conventional multi-point supine procedure, the posterior single-window cryoprobe trajectory (Figure 2) appeared associated with lower procedural discomfort, greater calmness, improved tolerability, and reduced procedural burden. According to caregiver observations, the child appeared significantly more relaxed and cooperative during the posterior single-window approach.

The approach facilitated direct visualization of the posterior muscular compartments and enabled ergonomic alignment between the ultrasound probe and cryoprobe trajectory (Figure 2).

## 3. Discussion

Previous studies emphasized the importance of selective targeting of tibial motor branches in the management of pediatric spastic equinovarus [1]. Similarly, recent cryoneurolysis literature increasingly supports the concept of dynamic and multimodal focal spasticity management [3,4,7,9,10].

The present report extends these concepts to a minimally invasive cryoneurolysis setting and proposes a simplified posterior single-window strategy for selected severe pediatric patients with complex deforming spasticity patterns. The report also highlights the potential relevance of procedural workflow, patient tolerability, and caregiver-centered outcomes in pediatric neurorehabilitation.

Conventional approaches derived from selective chemodenervation paradigms may offer high anatomical selectivity but frequently require multiple accesses and repeated probe repositioning maneuvers. In contrast, the proposed posterior single-window approach strategy appeared to simplify procedural workflow, reduce the number of skin punctures, improve procedural ergonomics, and reduce procedural burden in this single case.

A particularly relevant finding emerging from the present same-case comparison was the substantial difference in procedural tolerability between the two approaches. The posterior single-window strategy was completed using only minimal local anesthesia and required approximately 4 min, whereas the conventional three-point supine approach required minimal midazolam sedation because of discomfort and poor procedural tolerance and lasted approximately 20 min.

This difference may be particularly relevant in pediatric neurorehabilitation settings where procedural compliance, emotional stress, repeated sedation exposure, and caregiver burden may strongly influence the feasibility and acceptability of repeated focal spasticity interventions. In severe pediatric cerebral palsy, technical simplification and reduction in procedural fragmentation may therefore represent clinically meaningful advantages beyond pure anatomical selectivity.

Recent technical observations in cryoneurolysis procedures suggested that altered functional anatomy in severe chronic spasticity may represent an important procedural limitation and may require adaptive targeting strategies [12]. Similar concepts emerged in our previous technical experience regarding distal motor-selective targeting and sequential biomechanical release strategies during musculocutaneous cryoneurolysis for severe post-stroke upper-limb spasticity [12].

In the present case, the prone posterior approach appeared to facilitate a more ergonomic and simplified access to the gastrocnemius–soleus/tibialis posterior complex while reducing procedural fragmentation. The possibility of sequentially targeting multiple motor branches through a single posterior window may represent an important practical advantage in pediatric patients with severe deforming patterns.

In severe pediatric cerebral palsy, clinically meaningful outcomes frequently extend beyond restoration of gait and may include facilitation of hygiene, improved positioning, easier dressing, reduction in stiffness, facilitation of rehabilitation handling, and reduction in caregiver burden [2]. In the present case, the observed reduction in MAS scores, improvement in passive ankle dorsiflexion, and reduction in semitendinosus-related hypertonia appeared clinically consistent with improved rehabilitation handling and caregiver-assisted mobilization. Although these observations remain exploratory because of the single-case design, they may support the hypothesis that simplified ultrasound-guided cryoneurolysis workflows could positively influence both biomechanical management and procedural tolerability in severe pediatric spasticity.

In the present case, caregivers perceived the clinical improvement as greater and more sustained compared with previous botulinum toxin treatments, particularly regarding flexibility, mobilization, and assisted standing activities.

The present report may also support the concept that ultrasound-guided cryoneurolysis should not be interpreted exclusively as a neurodestructive procedure, but rather as a dynamic minimally invasive modulation strategy integrated within multidisciplinary neurorehabilitation management. In this context, reduction in focal overactivity may facilitate rehabilitation handling, orthotic positioning, assisted standing, and caregiver-assisted mobilization even in patients with severe chronic motor impairment.

These observations should be interpreted as hypothesis-generating and cannot establish clinical superiority. The present report describes a single-case technical comparison and no definitive conclusions regarding efficacy or superiority can be drawn.

Additional limitations include the absence of quantitative gait analysis, objective biomechanical measurements, electrophysiological evaluation, and standardized pain scales. Further prospective studies are needed to evaluate reproducibility, procedural duration, caregiver satisfaction, procedural discomfort, sedation requirements, and long-term functional outcomes.

Future investigations may help clarify whether simplified posterior single-window strategies could improve feasibility and tolerability of repeated cryoneurolysis procedures in children with severe spastic cerebral palsy requiring multimodal focal spasticity management.

### Limitations

The present report describes a single-patient technical comparison and therefore no definitive conclusions regarding efficacy, superiority, or reproducibility can be drawn.

The same-patient comparison is subject to inherent methodological limitations, including potential operator-dependent bias, sequence-related bias, observer bias, and expectation bias. The procedures were not randomized or blinded, and the qualitative comparison of tolerability was based on clinical observation and caregiver report rather than validated procedural discomfort or satisfaction scales.

Several additional limitations should be acknowledged. Quantitative gait analysis, objective biomechanical measurements, electrophysiological assessment, and standardized procedural discomfort scales were not available. Similarly, no formal quantitative caregiver burden assessment was performed.

Similarly, patient satisfaction and caregiver-perceived procedural tolerability were based on clinical and observational assessment rather than validated satisfaction scales.

Although clinically meaningful improvements in positioning, hygiene management, passive mobilization, assisted standing, and rehabilitation handling were observed, the outcomes mainly relied on clinical evaluation and caregiver-reported observations.

Further prospective investigations involving larger pediatric populations are required to evaluate reproducibility, long-term efficacy, procedural tolerability, sedation requirements, caregiver-centered outcomes, and potential integration with multidisciplinary spasticity management strategies.

## 4. Conclusions

This single-patient technical case report suggests that a posterior single-window ultrasound-guided cryoneurolysis approach may be technically feasible for selected children with severe spastic equinovarus. In this case, the approach was associated with shorter procedural duration, fewer access points, absence of sedation, and better qualitative procedural tolerability compared with a conventional multi-point supine strategy.

These findings should be interpreted cautiously because they derive from one patient and a short 3-month follow-up. No definitive conclusions regarding efficacy, superiority, reproducibility, or long-term safety can be drawn. Further prospective studies using standardized outcome measures are required to confirm whether this simplified procedural workflow may be useful in pediatric focal spasticity management.

## Figures and Tables

**Table 1:** Technical and procedural comparison between the posterior single-window cryoneurolysis approach and the conventional multi-point supine strategy.

Parameter	Posterior Single-Window	Conventional Multi-Point Supine Strategy
Patient position	Prone	Supine
Number of access points	One	Three
Main targets	Gastrocnemius, soleus, motor branchestibialis posterior-related motor branches	Soleus, tibialis posterior-related branches, lateral gastrocnemius motor branches
Probe repositioning	Minimal	Repeated
Local anesthesia	Approximately 1 mL of 2% lidocaine	Local anesthesia plus sedation
Sedation	Not required	Minimal conscious sedation with midazolam
Procedural duration	Approximately 4 min	Approximately 20 min
Tolerability	High	Lower
Immediate adverse events	None	None

## Data Availability

No new datasets were generated or analyzed during the preparation of this case report. Additional clinical information supporting the findings of this report is available from the corresponding author upon reasonable request, subject to the protection of patient confidentiality.
